# Association between neutrophil percentage-to-albumin ratio and all-cause mortality in patients with sepsis: A retrospective cohort study

**DOI:** 10.1097/MD.0000000000045980

**Published:** 2025-11-21

**Authors:** Guowei Zhu, Qian Cao, Jinming Liu, Shu Yang, Minmin Zhu, Xiao Liang

**Affiliations:** a Wuxi School of Medicine, Jiangnan University, Wuxi, Jiangsu Province, China; b Department of Anesthesiology, Jiangnan University Medical Center (Wuxi No. 2 People’s Hospital), Wuxi, Jiangsu Province, China; c Department of Anesthesiology, The Affiliated Yixing Hospital of Jiangsu University, Yixing, Jiangsu Province, China.

**Keywords:** all-cause mortality, medical information mart for intensive care, neutrophil percentage-to-albumin ratio, sepsis

## Abstract

Sepsis is a life-threatening syndrome caused by an imbalanced host response to infection and remains one of the leading causes of death worldwide. The neutrophil percentage-to-albumin ratio (NPAR) has recently emerged as a novel biomarker that integrates information on systemic inflammation and nutritional status. However, its role in predicting outcomes among septic patients has yet to be fully clarified. This study aimed to investigate the association between NPAR and all-cause mortality at 28-day, 90-day, and 365 day in patients diagnosed with sepsis. Using the medical information mart for intensive care-IV v3.1 database, we identified 6242 patients meeting the Sepsis-3.0 definition. Participants were categorized into tertiles based on NPAR values (*Q*_1_ < 23.8; 23.8 ≤ *Q*_2_ < 30.1; *Q*_3_ ≥ 30.1). Multivariable Cox proportional hazards regression was employed to analyze associations between NPAR and mortality. Restricted cubic spline models assessed nonlinear trends, while receiver operating characteristic curves evaluated discriminative ability. Kaplan–Meier survival analyses were performed to visualize survival differences across NPAR groups. Patients in the highest NPAR tertile (*Q*_3_) exhibited significantly greater disease severity and higher mortality at 28 days (34.03% vs 22.28%), 90 days (43.54% vs 28.93%), and 365 days (52.33% vs 37.72%) compared to those in the lowest tertile (*Q*_1_; all *P* < .001). After adjusting for confounders, elevated NPAR remained an independent predictor of mortality at 28 days (HR = 1.22, 95% CI: 1.06–1.39), 90 days (HR = 1.24, 95% CI: 1.10–1.40), and 365 days (HR = 1.21, 95% CI: 1.09–1.35). Restricted cubic spline analyses revealed a U-shaped nonlinear relationship between NPAR and mortality (*P* for nonlinearity < .001). Integrating NPAR with the sequential organ failure assessment score significantly enhanced predictive accuracy compared to sequential organ failure assessment alone (area under the curve: 0.624 vs 0.599, *P* < .001). Subgroup analysis indicated a more pronounced association in patients with congestive heart failure (*P* for interaction = .002). High NPAR levels are independently associated with increased short- and long-term mortality in sepsis. Given its simplicity and cost-effectiveness, NPAR could be a useful marker for early risk assessment and clinical decision-making in septic populations.

## 1. Introduction

Sepsis is a life-threatening condition characterized by organ dysfunction resulting from a dysregulated host response to infection. Its core pathophysiological feature is an imbalance between pro-inflammatory and compensatory anti-inflammatory responses induced by infection, ultimately leading to widespread physiological disturbances and multi-organ dysfunction. Sepsis typically has an acute onset and rapid progression, posing a significant threat to patient outcomes once it occurs.^[1]^ According to global estimates, approximately 49 million cases of sepsis occur each year, resulting in about 11 million deaths – accounting for nearly 19% of all global deaths. In intensive care units, sepsis is responsible for 30% to 50% of all deaths.^[2]^ Although modern critical care medicine has made significant progress, sepsis continues to pose a serious global public health challenge that demands urgent attention from healthcare systems worldwide.

Given the crucial importance of early identification and risk assessment in improving sepsis prognosis, clinical researchers have focused on identifying simple and reliable biomarkers for effective patient stratification. Numerous studies have shown that the degree of inflammatory response and immune dysregulation are key determinants of clinical outcomes in sepsis.^[3]^ Accordingly, a variety of hematologic inflammation-related markers – such as C-reactive protein (CRP), procalcitonin (PCT) and tumor necrosis factor-alpha (TNF-α) – are widely used in clinical prognosis assessment.^[4,5]^ However, these conventional biomarkers often suffer from limitations such as low specificity, high cost, or unstable dynamic changes.

The neutrophil percentage-to-albumin ratio (NPAR) is an emerging composite inflammatory marker that has attracted increasing attention due to its accessibility, low cost, and lack of need for additional testing procedures. NPAR combines the neutrophil percentage, which reflects acute inflammation, with serum albumin levels, an indicator of both inflammation and nutritional reserve, thereby providing a multi-dimensional view of the host’s inflammatory and immune status.^[6]^ Previous studies have demonstrated that NPAR has good prognostic predictive value in various critical illness contexts, including atrial fibrillation, acute ischemic stroke, cardiogenic shock, and acute kidney injury, suggesting its broad applicability across complex clinical scenarios.^[7–10]^

Nevertheless, current evidence on the role of NPAR in sepsis remains limited. Gong et al have preliminarily explored its predictive utility in patients with severe sepsis and septic shock, showing that elevated NPAR is associated with increased risk of in-hospital mortality.^[11]^ However, their study had a small sample size and was confined to a specific disease stage or population, lacking a systematic evaluation of all-cause mortality in a broader sepsis cohort. To fill this research gap, this study utilized the newly available medical information mart for intensive care IV (MIMIC-IV, version 3.1) database,^[12]^ which provides a large sample size and high-quality clinical data, to comprehensively evaluate the association between NPAR and 28-day, 90-day, and 365-day all-cause mortality in patients with sepsis. The aim is to provide a practical scientific basis for early risk stratification and personalized interventions in sepsis management, ultimately contributing to improved clinical outcomes and optimized treatment strategies.

## 2. Materials and methods

### 2.1. Data source

This study was designed as a retrospective cohort analysis utilizing data extracted from the MIMIC-IV v3.1 database.^[12]^ The MIMIC-IV database is a publicly available critical care dataset developed through a collaboration between the Massachusetts Institute of Technology, Beth Israel Deaconess Medical Center, and Philips Healthcare. It contains comprehensive clinical data from patients admitted between 2001 and 2019, including demographics, hospital admissions, laboratory test results, vital signs, medications, diagnostic codes, and nursing notes. This rich and diverse dataset serves as a valuable resource for conducting retrospective observational studies in critical care settings.

To protect patient privacy, all personal identifiers in the database have been removed and replaced with randomly generated codes through a de-identification process. As the data were de-identified and publicly available, neither individual informed consent nor institutional review board approval was necessary for this study. The authors of this study have completed the required data usage training provided by the collaborating institutions (certification number: 66989309) and are authorized to access and extract data from the database.

### 2.2. Patients

Inclusion criteria for this study were as follows: patients diagnosed with sepsis according to the Sepsis-3.0 definition, defined by a sequential organ failure assessment (SOFA) score ≥ 2,^[1]^ for patients with multiple intensive care unit (ICU) admissions, only data from the first ICU stay were included. Patients were excluded if their ICU stay was ≤24 hours or if data on neutrophil percentage or serum albumin were missing.

### 2.3. Data extraction and processing

This study utilized Navicat Premium Lite 17 and Structured Query Language to extract relevant data from the MIMIC-IV v3.1 database. The collected data encompassed the following domains: demographics: age and gender; comorbidities: including myocardial infarction, severe liver disease, congestive heart failure, peripheral vascular disease, cerebrovascular disease, chronic pulmonary disease, diabetes, renal disease, and malignant cancer; severity scores on the first day of ICU admission: acute physiology and chronic health evaluation III (APSIII), logistic organ dysfunction system (LODS), Oxford acute severity of illness score (OASIS), SOFA, Glasgow coma scale, simplified acute physiology score III (SAPSIII), and Charlson comorbidity index (CCI); vital signs on the first ICU day: heart rate, respiratory rate, mean blood pressure (MBP), temperature, and peripheral oxygen saturation (SpO₂); mechanical ventilation status during the ICU stay; laboratory tests on the first ICU day: bicarbonate, calcium, blood urea nitrogen (BUN), potassium, sodium, glucose, alanine aminotransferase (ALT), alkaline phosphatase (ALP), international normalized ratio (INR), aspartate aminotransferase (AST), total bilirubin, neutrophils, absolute monocytes, absolute lymphocytes, white blood cell (WBC), red cell distribution width (RDW), prothrombin time (PT), platelet count, hemoglobin, partial thromboplastin time (PTT), albumin, and anion gap. For continuous variables, implausible values (e.g., a neutrophil percentage of 0) were treated as missing. Variables with more than 20% missing data were excluded from further analysis. For those with <20% missingness, multiple imputation techniques were applied to handle the missing values, aiming to maintain data integrity and ensure the robustness of the analytical results.

### 2.4. Statistical analysis

The distribution of continuous variables was evaluated using the Shapiro–Wilk test. Due to the non-normal distribution of these variables, results were expressed as medians with interquartile ranges (IQR), and group comparisons were carried out using the Mann–Whitney *U* test. Categorical variables were summarized as counts and percentages, with group differences assessed via the chi-square test where appropriate. To investigate the relationship between NPAR and all-cause mortality at 28-day, 90-day, and 365 day among sepsis patients, Cox proportional hazards regression models were constructed. Two models were applied: model 1 included adjustments for age and gender; model 2 extended these adjustments to include comorbid conditions, laboratory values, severity scores, and use of mechanical ventilation. To prevent multicollinearity and ensure the robustness of the models, neutrophil percentage and albumin were excluded. The variance inflation factor was used to detect multicollinearity among continuous predictors, with those exceeding a variance inflation factor threshold of 5 removed from the final model.^[13]^ Additionally, restricted cubic spline (RCS) functions were employed to graphically depict the association between NPAR and mortality risk. The predictive utility of NPAR was further assessed using receiver operating characteristic (ROC) curve analysis. Subgroup analyses were also performed to examine the relationship between NPAR and mortality across various patient subsets. Additionally, we performed a sensitivity analysis using a multivariable Cox proportional hazards model, excluding patients with malignancies and severe liver disease to assess the robustness of the results. All statistical analyses were conducted using SPSS version 27.0 (IBM Corp., Armonk ) and R version 4.3.3, with a 2-sided *P*-value < .05 considered statistically significant.

## 3. Results

### 3.1. Baseline characteristics (n = 6242)

**Figure 1.**
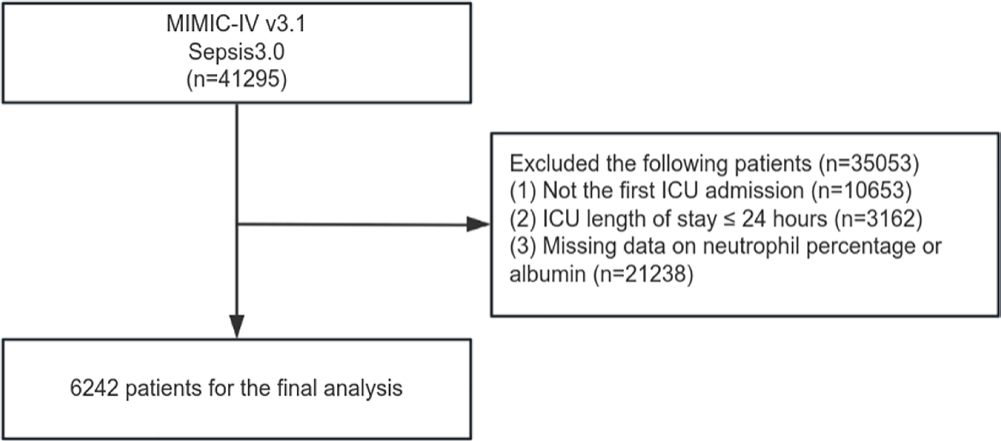
Research flowchart.

From the MIMIC-IV v3.1 database, a total of 41,295 sepsis patients were initially identified. Following the application of predefined inclusion and exclusion criteria, 6242 individuals were retained for the final analysis. The procedures for data preprocessing and selection are detailed in Figure 1. Participants were categorized into 3 groups based on NPAR tertiles: *Q*_1_ (<23.8), *Q*_2_ (23.8 ≤ NPAR < 30.1), and *Q*_3_ (≥30.1). Baseline characteristics are presented in Table 1. The median age of the included cohort was 65.79 years (IQR: 54.19–77.21), and 56.09% (3501/6242) were male. Compared with the lower NPAR tertiles, patients in the highest tertile (*Q*_3_) had significantly higher values for heart rate, respiratory rate, SOFA, CCI, APSIII, LODS, OASIS, SAPSII, BUN, ALP, INR, AST, total bilirubin, neutrophils, WBC, RDW, PT, platelet, and PTT. In contrast, they had lower values for MBP, SpO₂, bicarbonate, calcium, absolute monocytes, absolute lymphocytes, hemoglobin, and albumin. Furthermore, the *Q*_3_ group exhibited a higher prevalence of comorbidities, including myocardial infarction, congestive heart failure, peripheral vascular disease, cerebral vascular disease, chronic pulmonary disease, diabetes, renal disease, and malignant cancer. Moreover, patients in the *Q*_3_ group had longer ICU stays and significantly higher 28-day, 90-day, and 365-day mortality rates compared with those in the *Q*_1_ group.

**Table 1:** Baseline characteristics of the included patients.

Variables	Total (n = 6242)	*Q*_1_ (n = 2060)	*Q*_2_ (n = 2122)	*Q*_3_ (n = 2060)	*P*
Demographic					
Age, (yr)	65.79 (54.17, 77.21)	65.01 (53.83, 76.62)	66.08 (54.17, 77.85)	66.42 (54.72, 77.15)	.086
Gender, male (%)	3501 (56.09)	1208 (58.64)	1162 (54.76)	1131 (54.90)	.017
Vital signs					
Heart rate (bpm)	93.00 (80.00, 109.00)	91.00 (78.00, 107.00)	92.50 (79.00, 108.00)	97.00 (82.00, 112.00)	<.001
Mbp (mm Hg)	81.00 (69.00, 93.00)	84.00 (72.00, 97.00)	80.00 (69.00, 93.00)	78.00 (67.00, 90.00)	<.001
Resp rate (bpm)	20.00 (17.00, 25.00)	20.00 (16.00, 24.00)	20.00 (17.00, 25.00)	21.00 (17.00, 26.00)	<.001
Temperature (°C)	36.78 (36.44, 37.22)	36.78 (36.44, 37.22)	36.83 (36.44, 37.22)	36.78 (36.43, 37.17)	.004
Spo_2_ (%)	97.00 (94.00, 100.00)	97.00 (95.00, 100.00)	97.00 (94.00, 100.00)	97.00 (94.00, 100.00)	.007
Comorbidities, n (%)					
Myocardial infarct, n (%)	1131 (18.12)	401 (19.47)	401 (18.90)	329 (15.97)	.007
Severe liver disease, n (%)	807 (12.93)	255 (12.38)	277 (13.05)	275 (13.35)	.635
Congestive heart failure, n (%)	1974 (31.62)	645 (31.31)	737 (34.73)	592 (28.74)	<.001
Peripheral vascular disease, n (%)	624 (10.00)	175 (8.50)	231 (10.89)	218 (10.58)	.020
Cerebrovascular disease, n (%)	840 (13.46)	348 (16.89)	278 (13.10)	214 (10.39)	<.001
Chronic pulmonary disease, n (%)	1565 (25.07)	492 (23.88)	585 (27.57)	488 (23.69)	.005
Diabetes, n (%)	1956 (31.34)	638 (30.97)	709 (33.41)	609 (29.56)	.025
Renal disease, n (%)	1478 (23.68)	451 (21.89)	552 (26.01)	475 (23.06)	.005
Malignant cancer, n (%)	1011 (16.20)	337 (16.36)	260 (12.25)	414 (20.10)	<.001
Laboratory tests					
Bicarbonate (mEq/L)	21.00 (18.00, 25.00)	22.00 (19.00, 25.00)	22.00 (19.00, 25.00)	21.00 (17.00, 24.00)	<.001
Calcium (mg/dL)	8.20 (7.50, 8.70)	8.50 (8.00, 9.10)	8.20 (7.60, 8.70)	7.80 (7.20, 8.30)	<.001
Bun (mg/dL)	25.00 (16.00, 43.00)	22.00 (15.00, 37.00)	26.00 (16.00, 43.75)	28.00 (16.00, 50.00)	<.001
Potassium (mg/dL)	4.20 (3.70, 4.70)	4.20 (3.70, 4.70)	4.20 (3.70, 4.80)	4.10 (3.70, 4.70)	.056
Sodium (mg/dL)	138.00 (135.00, 141.00)	138.00 (135.00, 141.00)	138.00 (135.00, 141.00)	138.00 (134.00, 141.00)	<.001
Glucose (mg/dL)	132.00 (105.00, 178.00)	131.00 (105.00, 175.00)	134.50 (107.00, 181.00)	130.00 (102.00, 178.00)	.010
ALT (IU/L)	32.00 (18.00, 76.00)	28.00 (17.00, 64.00)	34.00 (18.00, 92.00)	34.00 (18.00, 78.00)	<.001
ALP (IU/L)	88.00 (63.00, 137.00)	80.00 (60.00, 112.00)	88.00 (63.00, 133.75)	102.00 (68.00, 172.25)	<.001
INR	1.40 (1.20, 1.70)	1.30 (1.10, 1.60)	1.30 (1.20, 1.70)	1.40 (1.20, 1.80)	<.001
AST (IU/L)	49.00 (26.00, 124.00)	44.00 (25.00, 105.00)	51.00 (28.00, 130.00)	52.00 (27.00, 134.00)	<.001
Bilirubin total (mg/dL)	0.80 (0.40, 1.80)	0.70 (0.40, 1.50)	0.70 (0.40, 1.80)	0.80 (0.40, 2.10)	<.001
Neutrophils (%)	82.50 (74.00, 88.20)	73.00 (60.45, 81.12)	83.80 (77.60, 88.50)	87.00 (82.00, 91.00)	<.001
Monocytes Abs (K/UL)	0.56 (0.31, 0.96)	0.58 (0.33, 0.99)	0.58 (0.33, 0.97)	0.53 (0.27, 0.93)	<.001
Lymphocytes Abs (K/UL)	0.93 (0.54, 1.48)	1.22 (0.73, 1.92)	0.87 (0.53, 1.34)	0.76 (0.43, 1.19)	<.001
WBC (K/UL)	12.10 (7.90, 17.90)	9.80 (6.38, 14.60)	12.50 (8.50, 17.80)	14.40 (9.60, 20.52)	<.001
RDW (%)	15.10 (13.80, 16.90)	14.60 (13.50, 16.40)	14.90 (13.80, 16.70)	15.70 (14.30, 17.40)	<.001
PT (s)	14.90 (13.00, 18.70)	14.00 (12.40, 17.40)	14.80 (13.00, 18.70)	15.80 (13.70, 19.62)	<.001
Platelet (K/UL)	184.00 (119.00, 262.00)	180.00 (117.00, 249.00)	185.00 (123.00, 260.00)	185.00 (114.00, 283.00)	.002
Hemoglobin (g/dL)	10.50 (8.90, 12.30)	11.40 (9.40, 13.20)	10.70 (9.00, 12.20)	9.70 (8.30, 11.20)	<.001
PTT (s)	31.90 (27.80, 40.10)	31.50 (27.30, 39.50)	31.40 (27.60, 39.10)	32.90 (28.50, 41.52)	<.001
Albumin (g/dI)	3.00 (2.50, 3.40)	3.60 (3.20, 3.90)	3.10 (2.90, 3.30)	2.40 (2.10, 2.70)	<.001
Aniongap (mEq/L)	15.00 (13.00, 19.00)	15.00 (13.00, 19.00)	15.00 (13.00, 19.00)	15.00 (12.00, 18.00)	<.001
Disease severity score					
SOFA	4.00 (2.00, 5.00)	3.00 (2.00, 5.00)	4.00 (2.00, 5.00)	4.00 (3.00, 6.00)	<.001
GCS	15.00 (15.00, 15.00)	15.00 (15.00, 15.00)	15.00 (15.00, 15.00)	15.00 (15.00, 15.00)	.733
CCI	5.00 (3.00, 7.00)	5.00 (3.00, 7.00)	5.00 (3.00, 8.00)	5.00 (3.00, 8.00)	.005
APSIII	54.00 (41.00, 72.00)	49.00 (36.00, 66.00)	53.00 (41.00, 67.75)	63.00 (48.00, 80.00)	<.001
LODS	6.00 (4.00, 8.00)	6.00 (4.00, 8.00)	6.00 (4.00, 8.00)	7.00 (5.00, 9.00)	<.001
OASIS	36.00 (30.00, 42.00)	34.00 (29.00, 41.00)	35.00 (30.00, 42.00)	37.00 (31.00, 43.25)	<.001
SAPSII	42.00 (33.00, 53.00)	39.00 (31.00, 51.00)	42.00 (33.00, 51.00)	46.00 (36.00, 56.00)	<.001
Intervention					
Ventilation, n (%)	5446 (87.25)	1769 (85.87)	1884 (88.78)	1793 (87.04)	.018
Outcomes					
ICU LOS (d)	3.96 (2.13, 8.40)	3.78 (1.99, 7.82)	3.85 (2.13, 8.23)	4.34 (2.31, 9.01)	<.001
28-d mortality, n (%)	1685 (26.99)	459 (22.28)	525 (24.74)	701 (34.03)	<.001
90-d mortality, n (%)	2209 (35.39)	596 (28.93)	716 (33.74)	897 (43.54)	<.001
365-d mortality, n (%)	2752 (44.09)	777 (37.72)	897 (42.27)	1078 (52.33)	<.001

ALP = alkaline phosphatase, ALT = alanine aminotransferase, APSIII = acute physiology score III, AST = aspartate aminotransferase, CCI = Charlson comorbidity index, GCS = Glasgow coma scale, INR = international normalized ratio, LODS = logistic organ dysfunction system, Mbp = mean blood pressure, OASIS = Oxford acute severity of illness score, PT = prothrombin time, PTT = partial thromboplastin time, RDW = red cell distribution width, SAPSIII = simplified acute physiology score III, SOFA = sequential organ failure assessment, SpO₂ = peripheral oxygen saturation, WBC = white blood cell.

### 3.2. Association between NPAR and all-cause mortality

We conducted multivariate analyses to investigate the association between NPAR and 28-day, 90-day, and 365-day all-cause mortality in patients with sepsis, and the results are presented in Table 2. When NPAR was analyzed as a continuous variable, elevated levels were significantly correlated with an increased risk of all-cause mortality across all statistical models (all *P*-values < .05). Furthermore, when NPAR was stratified into tertiles, with the lowest tertile serving as the reference, higher tertile levels were independently associated with greater risks of 28-day, 90-day, and 365-day all-cause mortality following adjustment for age, sex, and weight (tertile 3 vs tertile 1); HRs (95% CI): 1.61 (1.43–1.81), 1.65 (1.48–1.83), and 1.57 (1.43–1.73). In model 2, after further adjustment for age, gender, mechanical ventilation, myocardial infarction, severe liver disease, congestive heart failure, peripheral vascular disease, cerebrovascular disease, chronic pulmonary disease, diabetes, renal disease, malignant cancer, bicarbonate, calcium, BUN, potassium, sodium, glucose, ALT, ALP, INR, AST, total bilirubin, monocytes (absolute), lymphocytes (absolute), WBC, RDW, platelet, hemoglobin, PTT, anion gap, weight, heart rate, MBP, respiratory rate, temperature, SpO_2_, SOFA, Glasgow coma scale, CCI, APSIII, LODS, and OASIS scores, this association remained statistically significant. Higher NPAR was independently associated with increased risk of 28-day, 90-day, and 365-day all-cause mortality (tertile 3 vs tertile 1: HR, 95% CI: 1.22 (1.06–1.39), 1.24 (1.10–1.40), and 1.21 (1.09–1.35), respectively). In addition, the *P*-values for all trends were statistically significant (*P* < .05).

**Table 2:** Cox proportional hazard ratios for 28-day, 90-day and 365-day all-cause mortality.

	Non-adjusted		Model 1		Model 2	
	HR (95% CI)	*P*-value	HR (95% CI)	*P*-value	HR (95% CI)	*P*-value
28-d mortality						
NPAR	1.02 (1.02–1.03)	<.001	1.02 (1.02–1.03)	<.001	1.01 (1.00–1.01)	.012
Tertiles						
<23.8	1.00 (Reference)		1.00 (Reference)		1.00 (Reference)	
⩾23.8, <30.1	1.12 (0.99–1.27)	.086	1.10 (0.97–1.24)	.150	1.03 (0.91–1.17)	.644
≥30.1	1.62 (1.44–1.82)	<.001	1.61 (1.43–1.81)	<.001	1.22 (1.06–1.39)	.005
*P* for tread		<.001		<.001		.004
90-d mortality						
NPAR	1.02 (1.02–1.03)	<.001	1.02 (1.02–1.03)	<.001	1.01 (1.00–1.01)	.003
Tertiles						
<23.8	1.00 (Reference)		1.00 (Reference)		1.00 (Reference)	
⩾23.8, <30.1	1.19 (1.07–1.32)	.002	1.17 (1.05–1.30)	.006	1.11 (0.99–1.24)	.080
≥30.1	1.65 (1.49–1.83)	<.001	1.65 (1.48–1.83)	<.001	1.24 (1.10–1.40)	<.001
*P* for tread		<.001		<.001		<.001
365-d mortality						
NPAR	1.02 (1.02–1.03)	<.001	1.02 (1.02–1.03)	<.001	1.01 (1.00–1.01)	.001
Tertiles						
<23.8	1.00 (Reference)		1.00 (Reference)		1.00 (Reference)	
⩾23.8, <30.1	1.15 (1.05–1.27)	.003	1.13 (1.03–1.25)	.012	1.08 (0.98–1.19)	.142
≥30.1	1.57 (1.43–1.73)	<.001	1.57 (1.43–1.73)	<.001	1.21 (1.09–1.35)	<.001
*P* for tread		<.001		<.001		<.001

Non-adjusted model: Adjust for none.

Model 1: Adjusted for age and gender.

Model 2: Adjusted for age, gender, ventilation, myocardial infarction, severe liver disease, congestive heart failure, peripheral vascular disease, cerebrovascular Disease, chronic pulmonary disease, diabetes, renal disease, malignant Cancer, bicarbonate, calcium, BUN, potassium, sodium, glucose, ALT, ALP, INR, AST, total bilirubin, absolute monocytes, absolute lymphocytes, WBC, RDW, platelet count, hemoglobin, PTT, anion gap, heart rate, MBP, respiratory rate, temperature, SpO_2_, SOFA, GCS, CCI, APSIII, LODS, and OASIS.

ALP = alkaline phosphatase, ALT = alanine aminotransferase, APSIII = acute physiology score III, AST = aspartate aminotransferase, CCI = Charlson comorbidity index, GCS = Glasgow coma scale, INR = international normalized ratio, LODS = logistic organ dysfunction system, Mbp = mean blood pressure, OASIS = Oxford acute severity of illness score, PT = prothrombin time, PTT = partial thromboplastin time, RDW = red cell distribution width, SAPSIII = simplified acute physiology score III, SOFA = sequential organ failure assessment, SpO₂ = peripheral oxygen saturation, WBC = white blood cell.

### 3.3. Nonlinear association between NPAR and all-cause mortality in sepsis patients

**Figure 2.**
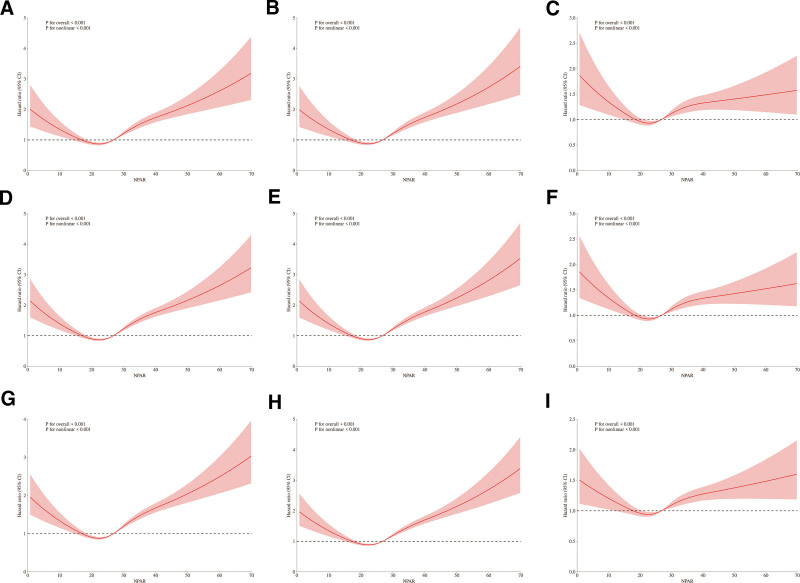
RCS regression analyses of NPAR with all-cause mortality at different time points. Panels (A–C) show associations with 28-day mortality (unadjusted, model 1-adjusted, and model 2-adjusted, respectively); panels (D–F) show associations with 90-day mortality; and panels (G–I) show associations with 365-day mortality. NPAR = neutrophil percentage-to-albumin ratio.

To further explore the relationship between NPAR and all-cause mortality, we conducted RCS analyses. As shown in Figure 2, both unadjusted and adjusted models revealed an U-shaped association between NPAR and the risk of 28-day, 90-day, and 365-day all-cause mortality. All tests for nonlinearity were statistically significant (*P* for nonlinearity < .001).

### 3.4. Survival analysis

**Figure 3.**
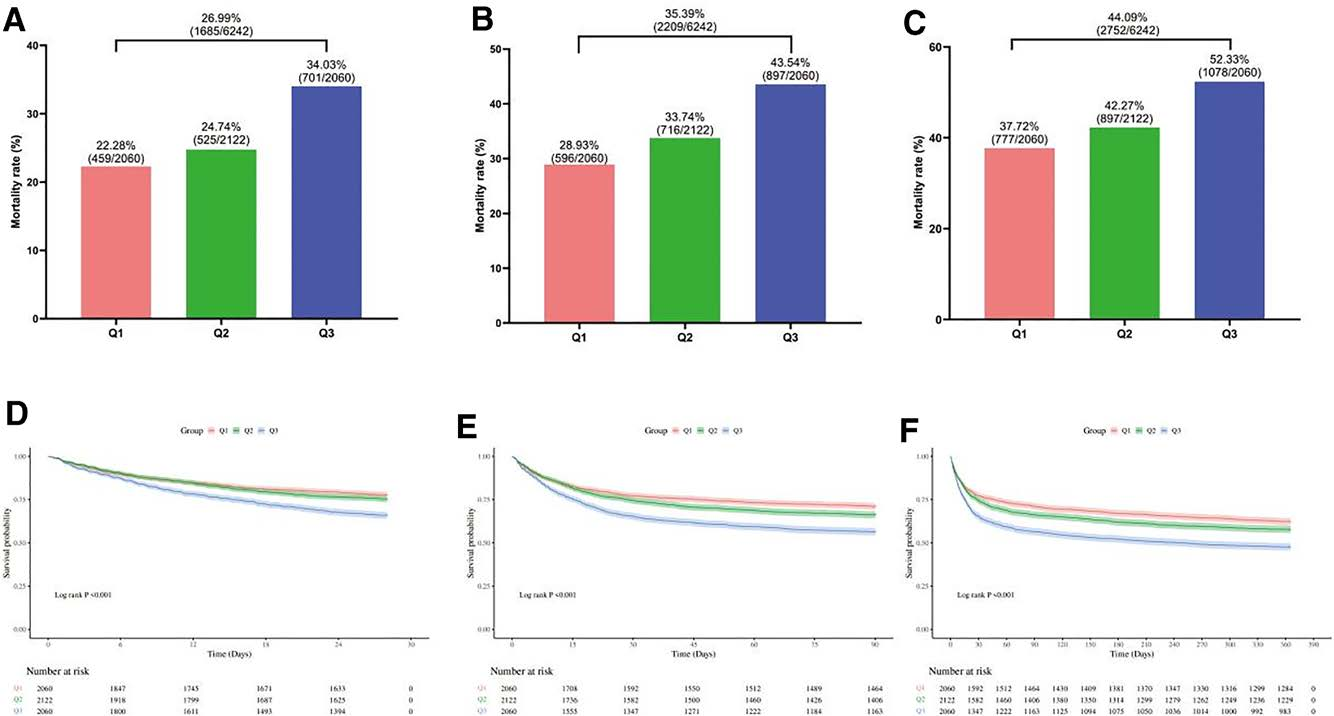
Mortality rates and Kaplan–Meier survival curves for the 3 NPAR groups. (A, D) 28-day mortality. (B, E) 90-day mortality. (C, F) 365-day mortality. NPAR = neutrophil percentage-to-albumin ratio.

Among the 6242 sepsis patients included in the analysis, 26.99% (1685/6242) died within 28 days, 35.39% (2209/6242) died within 90 days, and 44.09% (2752/6242) died within 1 year. Analyses across all subgroups demonstrated a significant upward trend in patient mortality with increasing NPAR. Kaplan–Meier survival analysis further confirmed that elevated NPAR was significantly associated with an increased risk of all-cause mortality in patients with sepsis (log-rank test, *P* < .001). This association remained statistically significant for 28-day, 90-day, and 365-day mortality. The results are shown in Figure 3.

### 3.5. Predictive performance of NPAR

**Figure 4.**
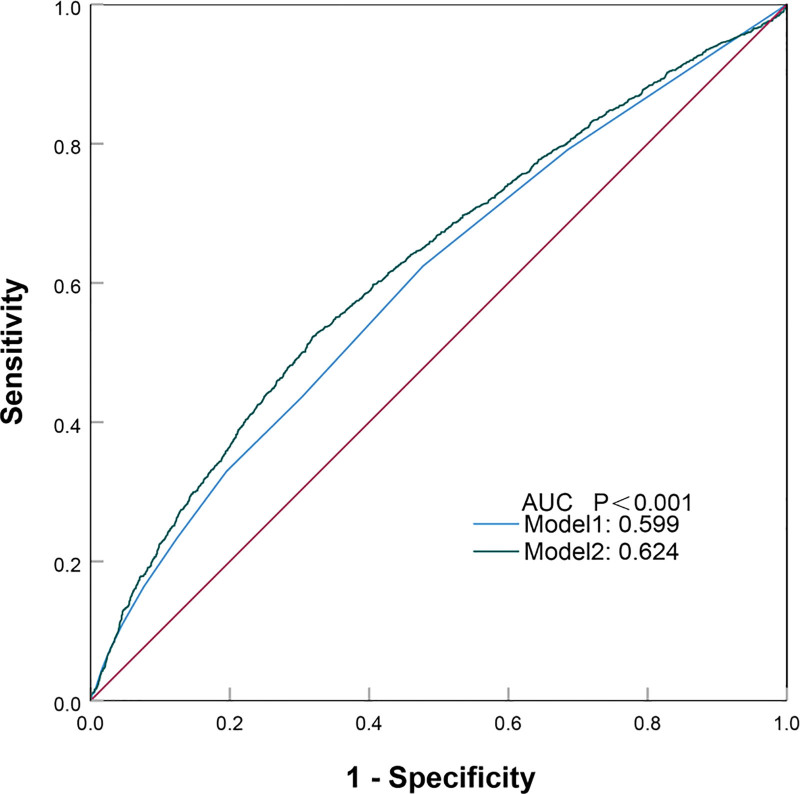
ROC curve predicting 28-day all-cause mortality in patients with sepsis. ROC = receiver operating characteristic.

To evaluate the predictive performance of NPAR for 28-day all-cause mortality risk, ROC curve analysis was conducted. Two models were constructed: model 1 included only the SOFA score, with an area under the curve of 0.599. Model 2 combined NPAR with SOFA, achieving an area under the curve of 0.624. According to the DeLong test, the improvement in predictive performance was statistically significant (*P* < .001). These results indicate that incorporating NPAR with SOFA significantly enhances the predictive ability of SOFA alone. The results are presented in Figure 4.

### 3.6. Subgroup analysis

**Figure 5.**
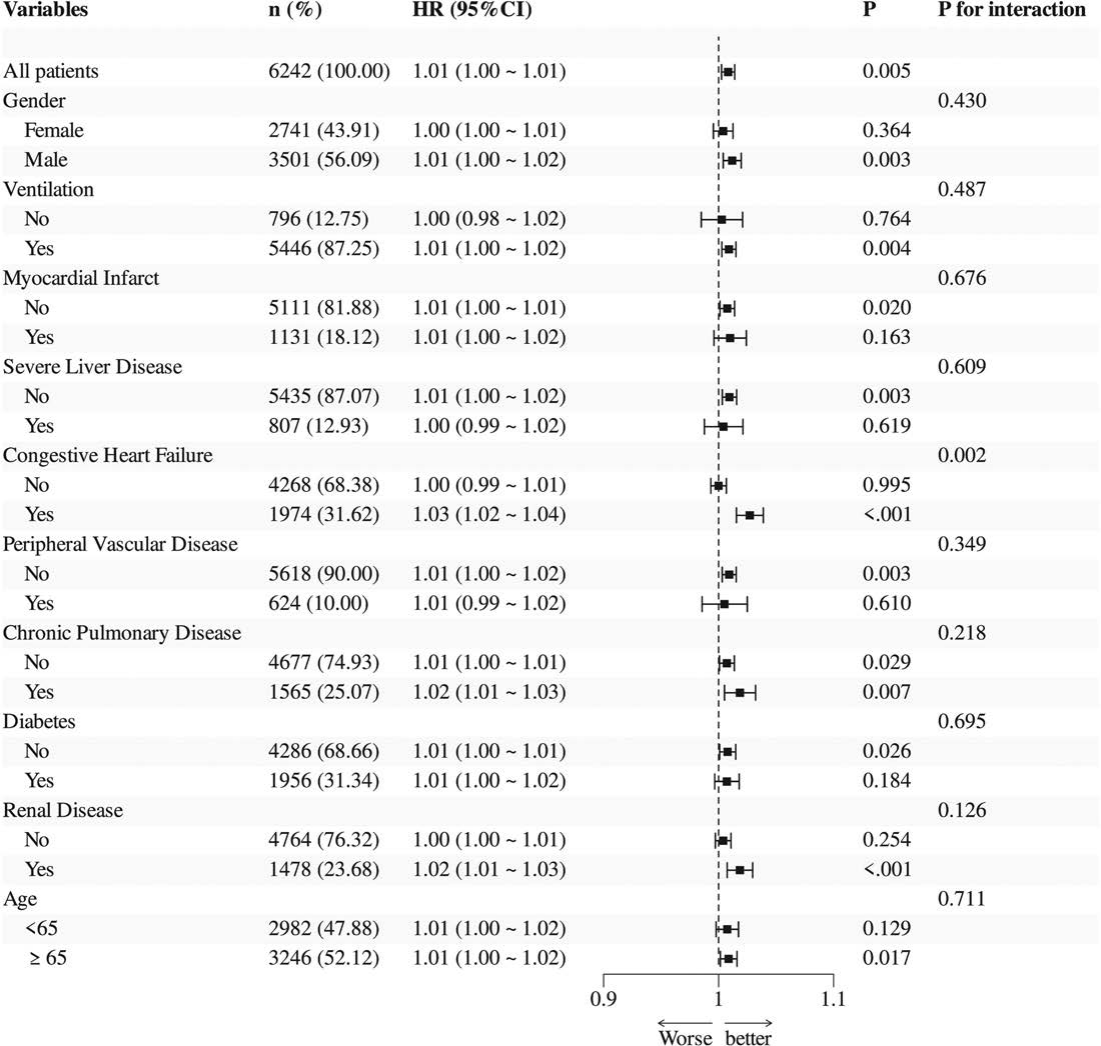
Subgroup analysis was performed in sepsis patients to investigate the association between the NPAR and 28-day all-cause mortality across different populations. NPAR = neutrophil percentage-to-albumin ratio.

To investigate the robustness and consistency of the link between NPAR and all-cause mortality among diverse clinical subgroups, we performed subgroup analyses based on age, gender, ventilation status, myocardial infarction, severe liver disease, congestive heart failure, peripheral vascular disease, chronic pulmonary disease, diabetes, and renal disease. In the overall population, NPAR was positively associated with all-cause mortality risk (HR [95% CI]: 1.01 [1.00–1.01]). Among the subgroups, a significant interaction was observed in the congestive heart failure group, with an interaction *P*-value of .002. No statistically significant interactions were observed across the remaining subgroups (all interaction *P*-values > .05), as illustrated in Figure 5.

### 3.7. Sensitivity analysis

To assess the robustness of the association between the NPAR and all-cause mortality in patients with sepsis, we performed sensitivity analyses by sequentially excluding patients with comorbid malignancies or severe liver disease. The results are summarized in Table 3. After excluding patients with malignancies, multivariate Cox regression analyses revealed that NPAR, when analyzed as a continuous variable, remained significantly and positively associated with the risk of all-cause mortality across all adjusted models (all HRs > 1, all *P* < .001). When NPAR was categorized into tertiles, with the lowest tertile (*Q*_1_) as the reference, the highest tertile (*Q*_3_) continued to demonstrate a significantly elevated risk of mortality (all *P* < .05). The hazard ratios (HRs) and 95% confidence intervals for 28-day, 90-day, and 365-day mortality were 1.21 (1.04–1.42), 1.28 (1.11–1.48), and 1.22 (1.08–1.39), respectively. Following the exclusion of patients with severe liver disease, highly consistent results were observed. NPAR as a continuous variable remained a significant predictor of mortality (all HRs > 1, all *P* < .05). In the analysis using tertiles, compared to *Q*_1_, *Q*_3_ was associated with significantly increased risks of 28-day, 90-day, and 365-day mortality, with HRs (95% confidence intervals) of 1.28 (1.10–1.49), 1.29 (1.13–1.47), and 1.26 (1.12–1.42), respectively (all *P* < .05). These sensitivity analysis results indicate that the positive association between NPAR and both short- and long-term all-cause mortality in sepsis patients remains robust across clinically important subgroups. The association is not substantially influenced by the presence of comorbidities such as malignancies or severe liver disease, thereby further supporting the reliability of our primary findings.

**Table 3:** Sensitivity analysis.

	Non-adjusted		Model 1		Model 2	
	HR (95% CI)	*P*-value	HR (95% CI)	*P*-value	HR (95% CI)	*P*-value
Exclude malignant tumors 28-d mortality						
NPAR	1.03 (1.02–1.03)	<.001	1.03 (1.02–1.03)	<.001	1.01 (1.00–1.02)	<.001
Tertiles						
<23.8	1.00 (Reference)		1.00 (Reference)		1.00 (Reference)	
⩾23.8, <29.7	1.10 (0.95–1.27)	.189	1.07 (0.93–1.23)	.337	1.03 (0.89–1.20)	.689
≥29.7	1.56 (1.36–1.78)	<.001	1.55 (1.36–1.77)	<.001	1.21 (1.04–1.42)	.017
90-d mortality						
NPAR	1.03 (1.02–1.03)	<.001	1.03 (1.02–1.03)	<.001	1.01 (1.00–1.02)	<.001
Tertiles						
<23.8	1.00 (Reference)		1.00 (Reference)		1.00 (Reference)	
⩾23.8, <29.7	1.19 (1.05–1.35)	.006	1.16 (1.02–1.31)	.021	1.12 (0.98–1.27)	.101
≥29.7	1.62 (1.44–1.83)	<.001	1.62 (1.44–1.83)	<.001	1.28 (1.11–1.48)	<.001
365-d mortality						
NPAR	1.02 (1.02–1.03)	<.001	1.03 (1.02–1.03)	<.001	1.01 (1.01–1.02)	<.001
Tertiles						
<23.8	1.00 (Reference)		1.00 (Reference)		1.00 (Reference)	
⩾23.8, <29.7	1.18 (1.06–1.32)	.003	1.15 (1.03–1.29)	.012	1.10 (0.98–1.23)	.120
≥29.7	1.52 (1.37–1.70)	<.001	1.53 (1.38–1.70)	<.001	1.22 (1.08–1.39)	.002
Exclude severe liver disease 28-d mortality						
NPAR	1.02 (1.02–1.03)	<.001	1.03 (1.02–1.03)	<.001	1.01 (1.01–1.02)	.005
Tertiles						
<23.8	1.00 (Reference)		1.00 (Reference)		1.00 (Reference)	
⩾23.8, <30.0	1.07 (0.93–1.23)	.322	1.04 (0.91–1.20)	.546	1.00 (0.87–1.16)	.975
≥30.0	1.68 (1.48–1.91)	<.001	1.66 (1.46–1.88)	<.001	1.28 (1.10–1.49)	.001
90-d mortality						
NPAR	1.03 (1.02–1.03)	<.001	1.03 (1.02–1.03)	<.001	1.01 (1.01–1.01)	.002
Tertiles						
<23.8	1.00 (Reference)		1.00 (Reference)		1.00 (Reference)	
⩾23.8, <30.0	1.16 (1.03–1.31)	.015	1.13 (1.01–1.27)	.050	1.09 (0.96–1.24)	.182
≥30.0	1.71 (1.52–1.91)	<.001	1.69 (1.51–1.89)	<.001	1.29 (1.13–1.47)	<.001
365-d mortality						
NPAR	1.02 (1.02–1.03)	<.001	1.02 (1.02–1.03)	<.001	1.01 (1.01–1.02)	<.001
Tertiles						
<23.8	1.00 (Reference)		1.00 (Reference)		1.00 (Reference)	
⩾23.8, <30.0	1.12 (1.01–1.25)	.030	1.09 (0.98–1.21)	.108	1.06 (0.95–1.19)	.299
≥30.0	1.62 (1.47–1.79)	<.001	1.61 (1.46–1.78)	<.001	1.26 (1.12–1.42)	<.001

Non-adjusted model: Adjust for none.

Model 1: Adjusted for age and gender.

Model 2: Adjusted for age, gender, ventilation, myocardial infarction, severe liver disease, congestive heart failure, peripheral vascular disease, cerebrovascular Disease, chronic pulmonary disease, diabetes, renal disease, malignant Cancer, bicarbonate, calcium, BUN, potassium, sodium, glucose, ALT, ALP, INR, AST, total bilirubin, absolute monocytes, absolute lymphocytes, WBC, RDW, platelet count, hemoglobin, PTT, anion gap, heart rate, MBP, respiratory rate, temperature, SpO_2_, SOFA, GCS, CCI, APSIII, LODS, and OASIS.

ALP = alkaline phosphatase, ALT = alanine aminotransferase, APSIII = acute physiology score III, AST = aspartate aminotransferase, CCI = Charlson comorbidity index, GCS = Glasgow coma scale, INR = international normalized ratio, LODS = logistic organ dysfunction system, Mbp = mean blood pressure, OASIS = Oxford acute severity of illness score, PT = prothrombin time, PTT = partial thromboplastin time, RDW = red cell distribution width, SAPSIII = simplified acute physiology score III, SOFA = sequential organ failure assessment, SpO₂ = peripheral oxygen saturation, WBC = white blood cell.

## 4. Discussion

This study conducted a large-scale retrospective cohort analysis of 6242 sepsis patients using the MIMIC-IV v3.1 database to systematically evaluate the association between the NPAR and all-cause mortality at 28, 90, and 365 days. The results demonstrated that elevated NPAR was significantly associated with increased short-term and long-term mortality risk, and this association remained statistically significant after multivariable adjustment. RCS regression further revealed a nonlinear U-shaped relationship between NPAR and mortality risk. Kaplan–Meier survival curves and ROC analyses also collectively supported the clinical potential of NPAR as a prognostic biomarker.

The findings of this study are consistent with those reported by Gong et al based on the MIMIC-III database, collectively confirming that NPAR is a robust predictor of mortality risk in sepsis patients.^[11]^ However, this study achieves significant advancements in several aspects: First, we adopted the Sepsis-3.0 criteria to define the patient cohort, thereby avoiding the heterogeneity associated with the older criteria and making the conclusions more aligned with current clinical practice. Second, using the updated MIMIC-IV v3.1 database, we validated the predictive efficacy of NPAR under contemporary sepsis management strategies. Most importantly, by employing RCS models, we revealed for the first time a nonlinear U-shaped relationship between NPAR and mortality, deepening the understanding of the association between NPAR and prognosis. In addition, we further validated the robustness of the findings through multiple statistical methods, including multivariable Cox regression, subgroup analyses, and sensitivity analyses.

The NPAR has emerged as a novel biomarker with significant prognostic value in sepsis. The systemic inflammatory response triggered by sepsis leads to neutrophil activation and a decline in serum albumin levels, both of which are closely associated with disease severity and mortality risk.^[14,15]^ Specifically, during the early phase of sepsis, activated neutrophils release large amounts of inflammatory mediators that further amplify the systemic inflammatory response. These mediators – such as tumor necrosis factor-α (TNF-α) and interleukin-6 (IL-6) – not only contribute to immune system dysregulation but also suppress hepatic albumin synthesis, directly resulting in hypoalbuminemia.^[16,17]^ Hypoalbuminemia itself is strongly linked to impaired immune function and poor clinical outcomes in septic patients. Elevated NPAR therefore reflects both intensified inflammation and the degree of immune suppression.^[18,19]^

Moreover, an increased NPAR is closely associated with organ dysfunction and higher mortality in sepsis. During the immunosuppressive phase of sepsis, low albumin levels impair neutrophil immune responses, reducing the body’s ability to combat infections.^[20]^ Concurrently, hepatic injury and disruption of intestinal barrier integrity promote albumin extravasation, exacerbating hypoalbuminemia. These processes amplify systemic inflammation and immune suppression, ultimately increasing the risk of death. Thus, NPAR, which integrates information from both neutrophil activation and albumin depletion, serves as a reliable predictor of mortality and multiple organ failure in sepsis.^[21,22]^

At the mechanistic level, elevated NPAR represents not only heightened inflammation but also underlying immune dysregulation. High NPAR values indicate persistent immune activation, which may evolve into immune exhaustion and secondary infections, both of which contribute to increased mortality.^[23,24]^ Therefore, NPAR is not merely a marker of disease severity but also a potential tool for early identification, risk stratification, and individualized management of patients with sepsis. This mechanism of interaction may partly explain the close relationship between elevated NPAR and increased mortality in patients with sepsis. As a simple, cost-effective, and easily obtainable laboratory marker, NPAR holds promising clinical value in risk stratification, therapeutic guidance, and prognostic assessment. Compared with complex scoring systems such as SOFA or APACHE II, NPAR offers more immediacy and operational convenience, particularly suitable for rapid assessment in resource-limited settings. Additionally, NPAR may serve as an indicator for monitoring treatment response, such as anti-inflammatory or nutritional interventions, thereby expanding its application in personalized therapeutic strategies.

Despite the robustness of our results based on a large sample size, several limitations should be noted. First, this was a single-center retrospective cohort study, which may be subject to selection and information bias, limiting the generalizability of the findings. Second, we calculated NPAR using only the initial laboratory data at ICU admission, thus unable to evaluate its dynamic changes over the disease course. Considering the fluctuating nature of sepsis, dynamic monitoring of NPAR might better capture its true relationship with prognosis. Furthermore, although multiple confounders were adjusted for, residual confounding from unmeasured variables, such as preexisting chronic diseases, treatment differences, or individual immune status, cannot be entirely excluded.

## 5. Conclusion

In summary, this study demonstrates that the NPAR, a composite marker reflecting both inflammatory and immune status, is significantly associated with increased short- and long-term all-cause mortality in patients with sepsis. Given its simplicity and low cost, NPAR holds promising clinical value and may serve as a useful tool for early identification, risk stratification, and dynamic management of sepsis. Nevertheless, further studies are warranted to validate its clinical utility and to explore strategies for enhancing its predictive performance.

## Acknowledgments

We are particularly grateful to MIMIC database for providing critical support to our data collection/analysis.

## Author contributions

**Conceptualization:** Guowei Zhu, Qian Cao, Jinming Liu, Shu Yang, Minmin Zhu.

**Data curation:** Qian Cao, Jinming Liu, Shu Yang.

**Formal analysis:** Guowei Zhu, Shu Yang.

**Investigation:** Guowei Zhu.

**Methodology:** Guowei Zhu.

**Supervision:** Minmin Zhu, Xiao Liang.

**Writing – original draft:** Guowei Zhu, Qian Cao, Jinming Liu.

**Writing – review & editing:** Shu Yang, Minmin Zhu, Xiao Liang.
